# Feasibility of randomizing Danish citizens aged 65–79 years to high-dose quadrivalent influenza vaccine vs. standard-dose quadrivalent influenza vaccine in a pragmatic registry-based setting: rationale and design of the DANFLU-1 Trial

**DOI:** 10.1186/s40814-022-01044-w

**Published:** 2022-04-21

**Authors:** Niklas Dyrby Johansen, Daniel Modin, Joshua Nealon, Sandrine Samson, Camille Salamand, Carsten Schade Larsen, Brian L. Claggett, Scott D. Solomon, Martin J. Landray, Gunnar H. Gislason, Lars Køber, Jens Ulrik Stæhr Jensen, Pradeesh Sivapalan, Lasse Skafte Vestergaard, Palle Valentiner-Branth, Tyra Grove Krause, Tor Biering-Sørensen

**Affiliations:** 1 Department of Cardiology, Copenhagen University Hospital - Herlev and Gentofte, Copenhagen, Denmark; 2 Department of Biomedical Sciences, Faculty of Health and Medical Sciences, University of Copenhagen, Copenhagen, Denmark; 3 Influenza Medical Evidence Generation, Sanofi Pasteur, Lyon, France; 4 School of Public Health, Li Ka Shing Faculty of Medicine, The University of Hong Kong, Pokfulam, Hong Kong, Special Administrative Region China; 5 Global Medical Affairs, Sanofi Pasteur, Lyon, France; 6 Clinical Department, Biostatistical Sciences, Sanofi Pasteur, Lyon, France; 7 Department of Clinical Medicine – Department of Infectious Diseases, Aarhus University Hospital, Aarhus, Denmark; 8 Cardiovascular Division, Brigham and Women’s Hospital, Harvard Medical School, Boston, MA USA; 9 Clinical Trial Service Unit and Epidemiological Studies Unit, Nuffield Department of Public Health, University of Oxford, Oxford, UK; 10 Big Data Institute, University of Oxford, Oxford, UK; 11 Department of Clinical Medicine, Faculty of Health and Medical Sciences, University of Copenhagen, Copenhagen, Denmark; 12 The Danish Heart Foundation, Copenhagen, Denmark; 13 The National Institute of Public Health, University of Southern Denmark, Copenhagen, Denmark; 14 Department of Cardiology, Copenhagen University Hospital – Rigshospitalet, Copenhagen, Denmark; 15 Respiratory Medicine Section, Department of Medicine, Copenhagen University Hospital – Herlev and Gentofte, Copenhagen, Denmark; 16 Department of Infectious Disease Epidemiology and Prevention, Statens Serum Institut, Copenhagen, Denmark

**Keywords:** Randomized controlled trial, Pragmatic, Influenza, Vaccine, Registry, Feasibility, Pneumonia

## Abstract

**Background:**

High-dose influenza vaccines provide better protection against influenza infection than standard-dose in persons aged 65 years and above; however, in most countries, high-dose vaccines are not widely implemented. Assessing the relative effectiveness of high-dose compared to standard-dose vaccines on hospitalizations and mortality would enable more robust public health and cost-effectiveness estimates. This study aims to investigate the feasibility of conducting a pragmatic randomized clinical trial in Denmark comparing high-dose to standard-dose vaccines utilizing existing vaccination infrastructure and the Danish nationwide health registries for data collection.

**Methods:**

The DANFLU-1 trial (NCT05048589) is a pragmatic, open-label, active-controlled randomized trial randomizing Danish citizens aged 65–79 years to either high-dose quadrivalent influenza vaccine or standard-dose quadrivalent influenza vaccine. The study utilizes the infrastructure of a private vaccination provider (Danske Lægers Vaccinations Service) for recruitment, inclusion, randomization, and vaccination. All collection of baseline and follow-up data including safety monitoring is performed centrally by the Department of Cardiology at Herlev and Gentofte Hospital, Copenhagen, Denmark using the Danish nationwide health registries. The study aims to include 40,000 participants during the 2021/2022 influenza season. The primary endpoints address feasibility and include the number of participants enrolled, randomization balance, and representativeness compared to the Danish general population. Relative vaccine effectiveness will also be assessed, however, this feasibility study is not powered for clinical outcomes and may be affected by the COVID-19 pandemic.

**Discussion:**

The DANFLU-1 study is investigating the feasibility of conducting a large-scale pragmatic clinical trial in Denmark utilizing existing infrastructure and the Danish nationwide registries. This will provide valuable insight, especially for potential future fully powered vaccine trials, but also for trials wishing to investigate other interventions.

**Trial registration:**

Clinicaltrials.gov: NCT05048589, registered September 17, 2021.

## Background

Influenza is a contagious, acute viral respiratory illness that may affect all age groups with presentations ranging from mild upper respiratory symptoms to severe cases resulting in hospitalization and/or death. In the northern and southern hemispheres, influenza causes seasonal epidemics of disease almost every winter. In tropical regions, influenza circulates continuously throughout the year, causing outbreaks more irregularly [1]. Global estimates of influenza-associated mortality and morbidity indicate that influenza causes approx. 290,000–650,000 respiratory deaths and approx. 5 million hospitalizations for lower respiratory tract infection annually [2, 3]. The elderly (aged 65 and above) and individuals with chronic healthcare conditions such as heart disease, lung disease, or diabetes display a heightened risk of influenza complications compared to younger healthy adults [4, 5]. Vaccination is the most effective method for reducing the incidence of influenza and influenza-related morbidity and mortality [6]. Seasonal vaccination of persons aged 65+ and other high risk groups is strongly recommended by the European Centre for Disease Control and numerous national governmental immunization guidelines; in the USA, the Centers for Disease Control and Prevention additionally recommends vaccination for all persons aged ≥ 6 months without contraindications [4, 5].

Previous studies have shown that antibody response and protection elicited by the standard formulation influenza vaccine are reduced for individuals aged 65 years or older as compared to younger adults [7, 8]. A high-dose (HD) influenza vaccine formulation (Fluzone® High-Dose), containing 60 μg of antigen from each influenza strain included in the vaccine, has been shown in a US randomized controlled trial (RCT) to reduce the incidence of influenza by approx. 25% with no increase in serious adverse events compared to standard-dose (SD) vaccine formulations in persons aged 65+ [9]. Observational studies have also demonstrated better effectiveness of the HD influenza vaccine across multiple influenza seasons, in different populations, and in different settings versus SD influenza vaccines [10]. A recent cluster-randomized trial also found that the HD vaccine reduced respiratory-related hospital admissions in nursing home residents aged 65+ when compared to SD vaccines [11].

A quadrivalent formulation of the HD influenza vaccine (QIV-HD; Fluzone® High-Dose Quadrivalent; Efluelda®) was licensed for use in persons aged 65+ in Denmark in April 2020. In the Danish 2020/2021 influenza season, a trivalent HD vaccine was offered to citizens aged 85+ only, and in the 2021/2022 influenza season, the Danish government vaccination program has been expanded to offer QIV-HD vaccine to citizens aged 80 years or older (age limit changed to 82 years after the protocol was finalized). Therefore, in the 2021/2022 season, citizens aged 65–79 years are offered SD vaccines despite evidence indicating that the licensed HD vaccine could reduce morbidity and improve clinical outcomes in this population group. To date, no individually randomized studies have assessed the effect of HD vs SD influenza vaccine on the incidence of hospitalizations for influenza or pneumonia and mortality. There would be significant public health interest in quantifying the potential benefit of QIV-HD vs SD quadrivalent influenza vaccine (QIV-SD) with respect to clinically important and impactful outcomes such as hospitalization for influenza or pneumonia in the 65–79 age group, including as inputs for cost-effectiveness analyses to guide future vaccination policies.

RCTs serve as the gold standard for decision-making, but they are complex, costly, and their results can sometimes be difficult to generalize to real-world patient populations due to restrictive inclusion/exclusion criteria. However, nationwide healthcare databases provide the opportunity to combine the gold standard of individual randomization—thereby providing unbiased results from which causal conclusions can be inferred—with representative real-world patient populations and pragmatic data collection [12]. With this approach, rigorous randomization of treatment allocation is integrated with existing data-collection platforms which may be used to collect baseline and follow-up information, greatly reducing complexity and costs. In Denmark, nationwide administrative registries enable effective collection of such information for all persons residing in Denmark [13, 14]. This is highly desirable in studies comparing one vaccine to another since these studies involve the estimation of relatively small effect sizes as compared to placebo-controlled studies, therefore requiring large sample sizes.

### Purpose

This pragmatic pilot RCT was designed to test the feasibility of randomizing a representative sample of Danish citizens aged 65–79 years to QIV-HD or QIV-SD in the 2021/2022 influenza season in a registry-based setting utilizing existing vaccination infrastructure to inform the planning of future large-scale, fully powered RCTs.

## Study design and methods

### Design and organization

**Fig. 1:**
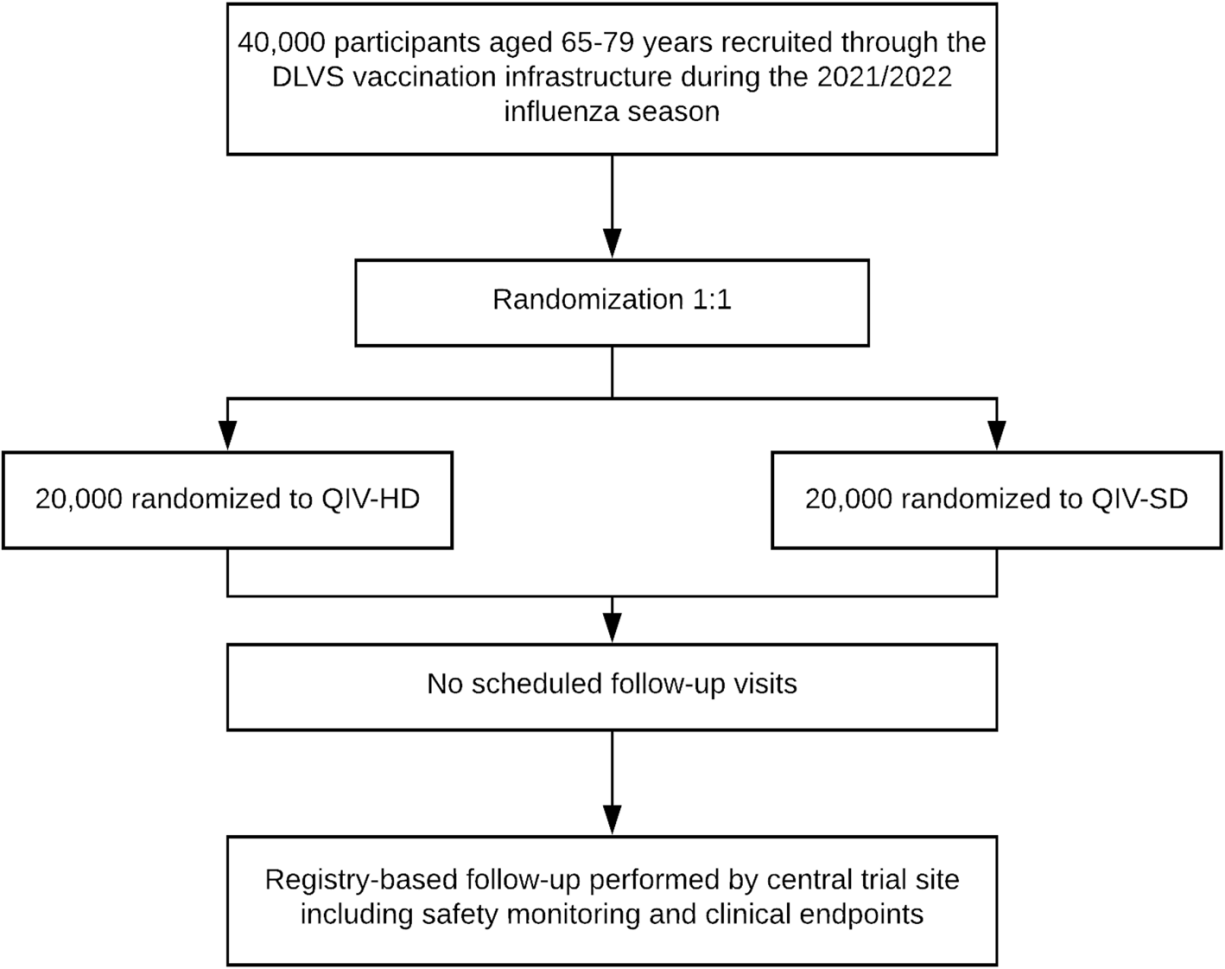
Study flowchart. *DLVS* Danske Lægers Vaccinations Service, *QIV*-*HD* high-dose quadrivalent influenza vaccine, *QIV*-*SD* standard-dose quadrivalent influenza vaccine

The study is a pragmatic, registry-based, open-label, active-controlled randomized trial serving as a pilot trial for a potential future fully powered trial. The study aims to randomize (1:1) 40,000 Danish citizens aged 65–79 years to QIV-HD or QIV-SD in the 2021/2022 influenza season (Fig. 1, Table 1).

**Table 1:** SPIRIT figure

Timepoint	Enrolment	Allocation and vaccine administration	Post-allocation	Close-out
	***Prior to or on Day 0***	***Day 0***	***3 months after vaccination***	***May 31, 2022***
**Enrolment**				
**Eligibility screen**	X			
**Informed consent**	X			
**Allocation**		X		
**Interventions**				
***QIV-HD***		X		
***QIV-SD***		X		
**Assessments**				
***Registry-based safety assessment***			X	
***Registry-based endpoint assessment***				X

*QIV*-*HD* high-dose quadrivalent influenza vaccine, *QIV*-*SD* standard-dose quadrivalent influenza vaccine

The study is conducted as a collaboration between (1) the Cardiovascular Non-Invasive Imaging Research Laboratory (CIRL) at the Department of Cardiology, Copenhagen University Hospital–Herlev and Gentofte, Copenhagen, Denmark; (2) the private vaccination provider Danske Lægers Vaccinations Service (DLVS); and (3) Statens Serum Institut (SSI). CIRL acts as the central trial coordination site and is responsible for study oversight, safety monitoring, and processing and analyzing registry data including endpoints. DLVS has over 20 years of experience in organizing vaccination sessions at both pop-up sites and stationary clinics and is responsible for recruitment, inclusion, randomization, vaccination, and initial data collection. The vaccination sessions are geographically dispersed across Denmark and participants are included at upwards of 1000 different vaccination sessions. SSI provides access to vaccination and microbiology databases and contributes expert knowledge on influenza vaccination.

Participants are randomized 1:1 to either QIV-HD or QIV-SD. Randomization is performed centrally using a computer-generated block randomization scheme.

The study aims to integrate a pragmatic randomized trial into routine vaccination practice by requiring only one study visit and performing registry-based follow-up without the need for additional visits or contacts with the study sites.

### Study population and sample size

Due to the pragmatic nature of the study, the only inclusion criteria are that the participants are aged 65–79 years and that the informed consent form has been signed and dated. The only exclusion criterion is allergy/hypersensibility toward the vaccines used in the study. Potential contraindications (fever etc.) are evaluated as part of routine practice on the day of vaccination.

Potential participants will be recruited by sending invitation letters to citizens vaccinated in prior seasons as recorded in an existing DLVS database. In addition, broader website-based advertising is also utilized. The projected sample size of 40,000 was chosen to, in the opinion of the study team, place vaccine delivery and vaccination program infrastructure and systems under sufficient pressure to inform the feasibility and planning of a future fully powered study. A preliminary sample size calculation yielded a required study population of 208,675 participants for a fully powered study under the assumptions of a true relative vaccine effectiveness (rVE) of QIV-HD to QIV-SD of 15%, an overall attack rate of hospitalization for influenza and/or pneumonia of 0.86% in the QIV-SD group, alpha of 0.05, and power of 0.90. Thus, this present study is not powered for the assessment of any clinical endpoints.

### Vaccines

The QIV-HD vaccine (Fluzone® High-Dose Quadrivalent, Sanofi Pasteur) contains 60 mg of hemagglutinin (HA) antigen for each of the four strains included in the vaccine. For this pragmatic study, any approved QIV-SD vaccine can be used as the active comparator. Both vaccines contain the same 4 strains based on the recommendations of the World Health Organization for the 2021/2022 northern hemisphere influenza season.

### Data collection and data sources

Initial data collection is performed by DLVS, who registers the social security number and name of the subject, the signed informed consent, randomization allocation, and the name and batch number of the administered vaccine. The informed consent form is electronic with signatures written on a tablet. All initial data including the electronic informed consent are automatically transferred to a central study database administered by CIRL.

The Danish health system is publicly funded and renowned for its administrative registries recording clinical events. The registries are frequently used for observational research [15]. In collaboration with the Danish Health Data Authority, the social security numbers of each subject are used to link the study database to the Danish nationwide administrative health registries. The Danish National Patient Registry contains information on all in- and outpatient visits in the Danish public health system [13]. The Danish National Prescription Registry contains claimed prescriptions at all pharmacies in Denmark [16]. The Danish Civil Registration System contains information on date of birth, deaths, immigration, and emigration [14]. The Danish Registry of Causes of death can be used to retrieve specific causes of death [17]. All vaccinations including prior influenza vaccinations can be retrieved from the Danish Vaccination Register [18]. The Danish Microbiology Database contains results from routinely performed tests including influenza [19]. Several registries containing socioeconomic data including personal income, pension, unemployment, and sickness benefits enable accurate assessment of the sociodemographic characteristics of the study population [20]. Education status can be retrieved from the Population Education Register [21].

Utilizing information from the registries enables the central trial coordination site to easily obtain information on baseline comorbidities, medication use at baseline, demographics, and socioeconomics as well as monitor clinical events during follow-up for safety assessment and clinical endpoints. The classification codes and definitions (e.g., ICD-10 and ATC codes) used for baseline conditions, medication use at baseline, and clinical endpoints are prespecified and can be found in Tables 2, 3, and 4. Prespecified definitions of registry-collected events ensure a minimum of bias in the assessment of these variables.

**Table 2:** Prespecified definitions for baseline conditions

Condition	ICD-10/ATC codes	Diagnosis type	Inpatient/outpatient/minimum required length of stay/other criteria	Timeframe
Chronic obstructive pulmonary disease	J42-J44	A or B	Any	≤ 10 years prior to randomization
Asthma	J45	A or B	Any	≤ 10 years prior to randomization
Chronic lung disease	A15-A16, D860, E84, J42-J47, J84, Z942	A or B	Any	≤ 10 years prior to randomization
Diabetes (ICD-10)	E10-E14	A or B	Any	≤ 10 years prior to randomization
Diabetes (ATC)	A10	–	≥ 1 claimed prescription	≤ 180 days prior to randomization
Hypertension (ICD-10)	I10-I15	A or B	Any	≤ 10 years prior to randomization
Hypertension (ATC)	Diuretics: C03 β-blockers: C07 Calcium blockers: C08 Renin-angiotensin system inhibitors: C09	–	≥ 1 claimed prescription from ≥ 2 drug classes	≤ 180 days prior to randomization
Dyslipidemia (ATC)	C10	–	≥ 1 claimed prescription	≤ 180 days prior to randomization
Ischemic heart disease	I20-I25	A or B	Any	≤ 10 years prior to randomization
Myocardial infarction	I21	A or B	Any	≤ 10 years prior to randomization
Heart failure	I50	A or B	Any	≤ 10 years prior to randomization
Other cardiomyopathy	I42-I43	A or B	Any	≤ 10 years prior to randomization
Atrial fibrillation	I48	A or B	Any	≤ 10 years prior to randomization
Other arrhythmia	I44-I47, I49	A or B	Any	≤ 10 years prior to randomization
Valvular disease	I34-I37	A or B	Any	≤ 10 years prior to randomization
Pericarditis	I30-I31	A or B	Any	≤ 10 years prior to randomization
Endocarditis	I33, I38-I39	A or B	Any	≤ 10 years prior to randomization
Myocarditis	I40-I41	A or B	Any	≤ 10 years prior to randomization
Pulmonary heart disease	I26-I28	A or B	Any	≤ 10 years prior to randomization
Cerebrovascular disease	I60-I69	A or B	Any	≤ 10 years prior to randomization
Peripheral vascular disease	I70, I74	A or B	Any	≤ 10 years prior to randomization
Congenital heart disease	Q20-Q26	A or B	Any	≤ 10 years prior to randomization
Chronic cardiovascular disease	I20-I28, I34-I37, I42-I50, I60-I69, I70, I74, Q20-Q26	A or B	Any	≤ 10 years prior to randomization
Cancer	C00-C97 (not C44)	A or B	Any	≤ 10 years prior to randomization
Chronic kidney disease	E102, E112, E132, E142, I120, N02-N08, N11-N12, N14, N18-N19, N26, N158-N160, N162-N164, N168, M300, M313, M319, M321B, Q612-Q613, Q615, Q619, T858-T859, Z992	A or B	Any	≤ 10 years prior to randomization
Liver disease	B15-B19, K70-K77, C22, I982, Z944, D684C, Q618	A or B	Any	≤ 10 years prior to randomization
Immunodeficiency (ICD-10)	B20-B24, D80-D84, D89, O987, Z21, Z940-Z944, Z948A	A or B	Any	≤ 10 years prior to randomization
Immunodeficiency (ATC)	H02AB, L04	–	≥ 1 claimed prescription	≤ 180 days prior to randomization
Neurological/neuromuscular disease	F00-F03, G10-G14, G20-G23, G30-G32, G35-G37, G40-G41, G70-G73, G80-G83, G91-G95	A or B	Any	≤ 10 years prior to randomization
Dementia	F00-F03, G30, G311-G312,	A or B	Any	≤ 10 years prior to randomization
Rheumatic disease	M05-M06, M32-M34, M353	A or B	Any	≤ 10 years prior to randomization

All baseline conditions are defined from ICD-10 codes except for diabetes, hypertension, dyslipidemia, and immunodeficiency, where ATC codes are also used. *ICD* International Classification of Diseases, *ATC* anatomic therapeutic chemical

**Table 3:** Prespecified definitions for medication use at baseline

Medication	ATC codes	Number of prescriptions	Timeframe
Antithrombotics	B01	≥ 1 claimed prescription	≤ 180 days prior to randomization
Renin-angiotensin system inhibitor	C09	≥ 1 claimed prescription	≤ 180 days prior to randomization
Calcium blockers	C08	≥ 1 claimed prescription	≤ 180 days prior to randomization
Beta blockers	C07	≥ 1 claimed prescription	≤ 180 days prior to randomization
Diuretics	C03	≥ 1 claimed prescription	≤ 180 days prior to randomization
Aspirin	B01AC06	≥ 1 claimed prescription	≤ 180 days prior to randomization
Statins	C10AA	≥ 1 claimed prescription	≤ 180 days prior to randomization
Inhaled beta-2 agonists	R03A	≥ 1 claimed prescription	≤ 180 days prior to randomization
Inhaled anticholinergics	R03BB, R03AL01-07	≥ 1 claimed prescription	≤ 180 days prior to randomization
Inhaled glucocorticoids	R03BA, R03AK, R03AL08-09, R03AL11-12	≥ 1 claimed prescription	≤ 180 days prior to randomization
Insulin	A10A	≥ 1 claimed prescription	≤ 180 days prior to randomization
Non-insulin antidiabetic medication	A10B	≥ 1 claimed prescription	≤ 180 days prior to randomization
Systemic glucocorticoids	H02AB	≥ 1 claimed prescription	≤ 180 days prior to randomization
Immunosuppres-sants	L04	≥ 1 claimed prescription	≤ 180 days prior to randomization

Medication use at baseline will be assessed using ATC codes. *ATC* anatomic therapeutical chemical

**Table 4:** Prespecified definitions for clinical endpoints

Endpoint	ICD-10 codes	Diagnosis type	Inpatient/outpatient/minimum required length of stay/other criteria	Timeframe
Influenza and/or pneumonia hospitalization	J09-J18	A	Inpatient, at least 1 night	≥ 14 days after vaccination and up to May 31, 2022
Respiratory disease hospitalization	J00-J06, J09-J18, J20-J22, J40-J47, J80-J81, J85-J86, J96	A	Inpatient, at least 1 night	≥ 14 days after vaccination and up to May 31, 2022
Cardiovascular disease hospitalization	I11, I13, I20-I25, I30-I31, I33, I38-I42, I46-I50, I60-I69, I74	A	Inpatient, at least 1 night	≥ 14 days after vaccination and up to May 31, 2022
Cardio-respiratory disease hospitalization	I11, I13, I20-I25, I30-I31, I33, I38-I42, I46-I50, I60-I69, I74, J00-J06, J09-J18, J20-J22, J40-J47, J80-J81, J85-J86, J96	A	Inpatient, at least 1 night	≥ 14 days after vaccination and up to May 31, 2022
Any hospitalization	Any	Any	Inpatient, at least 1 night	≥ 14 days after vaccination and up to May 31, 2022
COVID-19 hospitalization	B342, B972	A	Inpatient, at least 1 night	≥ 14 days after vaccination and up to May 31, 2022
All-cause death	–	–	–	≥ 14 days after vaccination and up to May 31, 2022

All clinical endpoints are defined from ICD-10 codes. *ICD* International Classification of Diseases

### Outcomes

All outcomes are assessed using data collected from the Danish administrative health registries. The outcomes are primarily feasibility-oriented and include:

- Number of persons contacted by recruitment letter
- Number of participants included and randomized to QIV-HD or QIV-SD
- Agreement between randomization group and administered vaccine
- Assessment of baseline characteristics according to randomization group
- Comparison of baseline characteristics between study population and Danish general population aged 65-79 years on October 1, 2021

The study is not powered for assessing clinical endpoints. However, for feasibility assessment purposes, event rates and relative vaccine effectiveness (rVE) will be assessed for the following clinical outcomes:

- Hospitalization for influenza and/or pneumonia
- Hospitalization for any respiratory disease
- Hospitalization for any cardio-respiratory disease
- Hospitalization for any cardiovascular disease
- Hospitalization from any cause
- All-cause mortality
- Hospitalization for COVID-19

The clinical outcomes are specified further in Table 4. rVE will be calculated as:

rVE = (1-(C_HD_ /N_HD_)/(C_SD_/N_SD_)) × 100%

Where:

C_HD_: describes the number of endpoints occurring in the QIV-HD arm meeting the endpoint definition

N_HD_: describes the number of participants in the QIV-HD arm

C_SD_: describes the number of endpoints occurring in the QIV-SD arm meeting the endpoint definition

N_SD_: describes the number of participants in the QIV-SD arm

### Assessment of success

Since this is the first trial ever conducted in Denmark to use the setup and design described in this report, the study team found it impossible to devise specific criteria for success with meaningful numerical cutoffs. Significant uncertainties pertaining primarily to the obtainment of approvals for this novel trial design from the Danish authorities and obtaining real-time access to registry data for a drug trial existed, leaving the study team wondering whether it would even be possible to initiate the trial. Due to this, the assessment of success will rely on an overall evaluation of the conduct of the trial including the obtainment of approvals, recruitment rate and success, randomization agreement, and reliability with regards to the registry-based obtainment of baseline characteristics, safety events, and clinical endpoints. This overall evaluation will be used by the study team as part of the decision on whether to progress to a fully powered trial. Any specific issues encountered during this feasibility trial will be addressed in the design of a fully powered trial, in case the study team should decide to progress.

### Safety monitoring

For this trial studying two influenza vaccines already approved for use in the study population, safety monitoring is performed using a pragmatic, registry-based approach. Each subject is assessed for the occurrence of serious adverse events (SAEs) and serious adverse reactions (SARs) at approx. 3 months after vaccination using extracts from the Danish administrative registries. Each episode, primarily deaths and hospitalizations, is assessed for causality, seriousness, and expectedness by a medical doctor based on electronic medical record review. Events are reported to the authorities according to applicable rules and regulations.

### Statistical analysis

Purely descriptive outcomes such as number of persons contacted and recruited will be presented in raw numbers. The proportion of the number of participants recruited compared to the number of persons contacted will be calculated and presented along with a 95% confidence interval. The proportion of participants not receiving the correct intervention will be calculated for each randomization arm and presented along with 95% confidence intervals. In the comparison of baseline characteristics between the study population and the overall Danish general population aged 65–79 years, characteristics will be compared using methods appropriate for each characteristic. Continuous baseline variables will be summarized using means and standard deviations or medians and interquartile ranges. Binary or categorical variables will be compared using frequencies and proportions. Event rates for clinical endpoints will be presented as attack rates and incidence rates with 95% confidence intervals. rVE estimates will be presented along with 95% confidence intervals. *P* values < 0.05 will be considered statistically significant. rVE estimates will be considered statistically significant if the confidence interval does not include 0.

### Ethical statement

The study complies with the standards of the CONSORT statement and the principles of the Declaration of Helsinki and undergoes monitoring according to the International Conference on Harmonization guidelines for Good Clinical Practice. The study has been approved by the regional research ethics committee (H-21035316), the Danish Medicines Agency (2021-003170-31), and the data authority in the Capital Region of Denmark (P-2021-407). The study is registered in the European Union Clinical Trials Register (EudraCT no. 2021-003170-31) and on Clinicaltrials.gov (NCT05048589).

Both negative, positive, and inconclusive results will be submitted for publication in peer-reviewed journals. Authorship criteria will be based on Recommendations for the Conduct, Reporting, Editing, and Publication of Scholarly Work in Medical Journals by The International Committee of Medical Journal Editors. The results of the study will be published on Clinicaltrials.gov and in the EudraCT database.

### Funding

The study is funded by Sanofi Pasteur, who provides the 20,000 QIV-HD doses free of charge along with a financial contribution covering expenses related to the trial. The study design and protocol were developed in collaboration with the funder. The sponsor of the study is Tor Biering-Sørensen, who is the head of CIRL.

## Discussion

To our knowledge, this is the largest study ever conducted in Denmark combining data collection from the renowned Danish nationwide administrative health registries with key elements from a gold standard RCT design. This feasibility study will provide valuable insight, especially for a potential future fully powered QIV-HD vs. QIV-SD trial, but will also provide operational and regulatory lessons for trials of other interventions conducted within the Danish health system.

The implementation of registry-collected data from real-world clinical events and procedures into randomized clinical trials provides trial sponsors and funders with a powerful tool to increase the scope and reduce the costs of large-scale trials. Event data can be passively collected instead of requiring active review of medical records or contact with study participants, enabling capture of a greater number of rare or difficult-to-ascertain endpoints, which may otherwise have been infeasible. This could in turn reduce total drug and vaccine development cost, benefitting patients and health systems due to a potential lower cost of the market-ready drug. The approach allows for robust measurement of effectiveness across a large number of potential indications and could provide increased confidence in health economic analyses, treatment guidelines, and clinical practice decisions.

Danish registry data has previously primarily been used for observational studies [22]. A reassuring finding of these studies is the minimal risk of loss-to-follow up due to the universal nature of the Danish healthcare system, a theoretical risk of pragmatic studies in which participants are not actively followed over time. Observing outcomes through the public health system lowers costs and complexity, but, more importantly, improves generalizability by allowing streamlined conduct of nationwide clinical trials without the need to coordinate across different health service providers and allows almost any citizen partaking in annual influenza vaccination to be included in a clinical study. This feature increases the likelihood of the findings being generalizable to real-world clinical practice and eases the complexity of interpreting or extrapolating results into specific population groups who may be at heightened risk.

Assessment of clinical endpoints using registry data is dependent on reliable coding and classification of hospital contacts by treating clinicians, and while a large proportion of diagnoses in the Danish registries are highly validated with high positive predictive values [13, 23–26], this might introduce the risk of underreporting when compared with laboratory-confirmed endpoints following active surveillance from regular clinical trials. Our study includes cardiovascular endpoints for which adjudication is commonplace, but the value of this approach has recently been questioned. A post-hoc analysis from the ASCEND trial assessing aspirin and/or ω-fatty acids for prevention of cardiovascular events in people with diabetes showed a strong agreement between routinely collected data and adjudicated follow-up for serious vascular events, and using routine data yielded similar effect sizes compared with adjudicated follow-up [27]. Additionally, in reports from the SHIFT [28] and PARADISE-MI [29] heart failure medication trials, it has been shown that adjudication did not have a meaningful impact on the studies’ effect estimates when compared to “raw” investigator-reported events. Additionally, using registry-based endpoints might even improve external validity of the study results [30].

A potential limitation of this registry-based approach is the lack of active surveillance and systematic laboratory confirmation corresponding to the primary study endpoint [31]. These features of classical efficacy clinical trials guarantee a relatively high incidence rate of specific events corresponding to regulatory requirements [32, 33]. Pragmatic, registry-based studies are therefore currently most relevant for post-licensure assessment.

This study is conducted as an open-label trial reducing the complexity and documentation demands related to handling of the vaccines at the vaccination sites. This will not impact the feasibility-oriented endpoints and would be expected to introduce only minimal bias in a potential future study evaluating registry-based hard clinical endpoints such as deaths and hospitalizations with prespecified definitions, especially in a trial comparing two active treatments without a placebo arm.

Based on extensive experience with both high-dose and standard-dose influenza vaccines from clinical practice and large-scale phase 3 trials [9, 34], and many years of use of both vaccine types in routine practice, it was agreed with the authorities that only the collection of SAEs, specifically deaths and hospitalizations, would be required. This further strengthens the case for the registry-based design since all deaths and hospitalizations in the Danish public health system can easily be passively collected from registry extracts, once again reducing the cost and complexity associated with the trial.

## Conclusions

The DANFLU-1 study is investigating the feasibility of conducting a large-scale pragmatic clinical trial in Denmark utilizing existing infrastructure for recruitment, randomization, and vaccination and the Danish nationwide registries for collection of baseline and follow-up data. This study will provide valuable insight, especially for potential future fully powered vaccine trials, but also for trials wishing to investigate other interventions.

## Data Availability

Baseline and endpoint data from this trial stems from Danish administrative health registries, which are subject to Danish legislation and can only be made available to a third party under certain conditions. Please contact the corresponding author in case of any inquiries.

## Abbreviations

ATC: Anatomic therapeutic chemical
CIRL: Cardiovascular Non-Invasive Imaging Research Laboratory
DLVS: Danske Lægers Vaccination Service
HA: Hemagglutinin
HD: High-dose
ICD: International Classification of Diseases
QIV-HD: High-dose quadrivalent influenza vaccine
QIV-SD: Standard-dose quadrivalent influenza vaccine
RCT: Randomized controlled trial
rVE: Relative vaccine effectiveness
SAE: Serious adverse event
SAR: Serious adverse reaction
SD: Standard-dose
SSI: Statens Serum Institut
